# Supplementary material for: Long noncoding RNA SAM promotes myoblast proliferation through stabilizing Sugt1 and facilitating kinetochore assembly
Source: Nat Commun. 2020 Jun 1;11:2725. doi: 10.1038/s41467-020-16553-6 (PMC7264179; doi:10.1038/s41467-020-16553-6)
Supplement: Supplementary file 1 — Supplementary Information [file 41467_2020_16553_MOESM1_ESM.pdf]

Supplementary Information

Long noncoding RNA SAM promotes myoblast proliferation through stabilizing Sugt1 and facilitating kinetochore assembly

Li *et al*. 2020

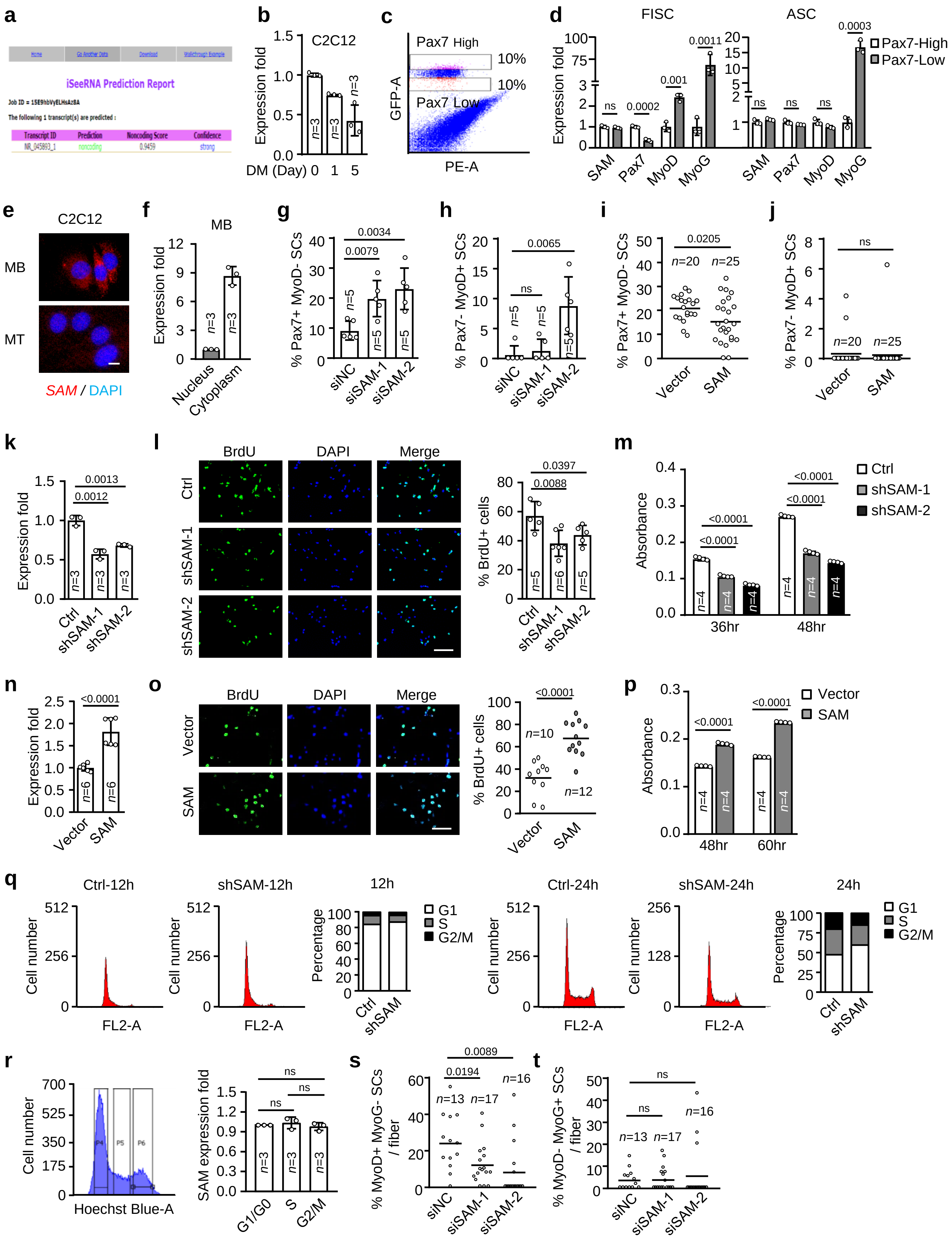

a
Home
Go Another Data
Download
Walkthrough Example
iSeeRNA Prediction Report
Job ID = 15E9hbVyELHsAz8A
The following 1 transcript(s) are predicted :
Transcript ID
Prediction
Noncoding Score
Confidence
NR_045893_1
noncoding
0.9459
strong
b
C2C12
Expression fold
1.5
1.0
0.5
0.0
n=3
n=3
n=3
DM (Day) 0 1 5
c
Pax7 High
10%
10%
Pax7 Low
GFP-A
PE-A
d
FISC
ASC
Expression fold
100
75
3
2
1
0
20
15
10
5
2
1
ns
0.0002
0.001
0.0011
ns
ns
ns
0.0003
Pax7-High
Pax7-Low
SAM
Pax7
MyoD
MyoG
SAM
Pax7
MyoD
MyoG
e
C2C12
MB
MT
SAM / DAPI
f
MB
Expression fold
12
9
6
3
0
n=3
n=3
Nucleus
Cytoplasm
g
% Pax7+ MyoD- SCs
40
30
20
10
0
0.0034
0.0079
n=5
n=5
n=5
siNC
siSAM-1
siSAM-2
h
% Pax7- MyoD+ SCs
20
15
10
5
0
0.0065
ns
n=5
n=5
n=5
siNC
siSAM-1
siSAM-2
i
% Pax7+ MyoD- SCs
50
40
30
20
10
0
0.0205
n=20
n=25
Vector
SAM
j
% Pax7- MyoD+ SCs
8
6
4
2
0
ns
n=20
n=25
Vector
SAM
k
Expression fold
1.5
1.0
0.5
0.0
0.0013
0.0012
n=3
n=3
n=3
Ctrl
shSAM-1
shSAM-2
l
BrdU
DAPI
Merge
Ctrl
shSAM-1
shSAM-2
% BrdU+ cells
80
60
40
20
0
0.0397
0.0088
n=5
n=6
n=5
Ctrl
shSAM-1
shSAM-2
m
Absorbance
0.4
0.3
0.2
0.1
0.0
Ctrl
shSAM-1
shSAM-2
<0.0001
<0.0001
<0.0001
<0.0001
n=4
n=4
n=4
n=4
n=4
n=4
36hr
48hr
n
Expression fold
2.5
2.0
1.5
1.0
0.5
0.0
<0.0001
n=6
n=6
Vector
SAM
o
BrdU
DAPI
Merge
Vector
SAM
% BrdU+ cells
100
80
60
40
20
0
<0.0001
n=10
n=12
Vector
SAM
p
Absorbance
0.3
0.2
0.1
0.0
Vector
SAM
<0.0001
<0.0001
n=4
n=4
n=4
n=4
48hr
60hr
q
Ctrl-12h
shSAM-12h
12h
Ctrl-24h
shSAM-24h
24h
Cell number
512
256
0
FL2-A
Cell number
512
256
0
FL2-A
Percentage
100
80
60
40
20
0
Ctrl
shSAM
G1
S
G2/M
Cell number
512
256
0
FL2-A
Cell number
256
128
0
FL2-A
Percentage
100
75
50
25
0
Ctrl
shSAM
G1
S
G2/M
r
Cell number
700
525
350
175
0
P4
P5
P6
Hoechst Blue-A
SAM expression fold
1.5
1.2
0.9
0.6
0.3
0.0
ns
ns
ns
n=3
n=3
n=3
G1/G0
S
G2/M
s
% MyoD+ MyoG- SCs / fiber
60
40
20
0
0.0089
0.0194
n=13
n=17
n=16
siNC
siSAM-1
siSAM-2
t
% MyoD- MyoG+ SCs / fiber
50
40
30
20
10
0
ns
ns
n=13
n=17
n=16
siNC
siSAM-1
siSAM-2

Supplementary Figure 1. *SAM* promotes C2C12 proliferation. (**a**) Predication of *SAM* coding potential by iSeeRNA (http://137.189.133.71/iSeeRNA/). (**b**) *SAM* expression at various time points in differentiating C2C12. DM, differentiation medium. (**c**) FACS sorting of FISCs from Pax7-nGFP mouse. Gatings for Pax7-High and Pax7-Low were indicated within total GFP population. (**d**) Relative gene expression in FISC and ASC from the above subpopulations ($n = 3$). (**e**) RNA-FISH for *SAM* transcripts (red) in C2C12 myoblasts (MB) and myotubes (MT). (**f**) *SAM* expression in cytoplasmic or nuclear fraction of MB. (**g**) The percentage of Pax7+MyoD- or (**h**) Pax7-MyoD+ cells in ASCs transfected with control (siNC) or *SAM* siRNA was quantified. (**i**) The percentage of Pax7+MyoD- or (**j**) Pax7-MyoD+ cells in ASCs transfected with a Vector or *SAM* expressing plasmid was quantified. (**k**) Stable knockdown of *SAM* in C2C12 cells by a shRNA. (**l**). The percentage of BrdU+ nuclei was quantified in the above cells. (**m**) MTT assay was performed at indicated time points in the above cells. (**n**) *SAM* expression in C2C12 transfected with a Vector or *SAM* expressing plasmid. (**o**) The percentage of BrdU+ nuclei was quantified in the above cells. (**p**) MTT assay was performed at indicated time points in the above cells. (**q**) sh*SAM* cells were synchronized by serum starvation for 24 h followed by cell cycle analysis at indicated time points after refeeding with medium. (**r**) Left: Distribution of myoblasts in the different phases of the cell cycle. P4, G1/G0 phase; P5, S phases; P6, G2/M phases. Right: *SAM* expression in the above subpopulations. (**s**) The percentage of MyoD+MyoG- or (**t**) MyoD-MyoG+ SCs on myofibers transfected with *SAM* siRNA was quantified. The data are represented as mean ± SD in **b, d, f-p** and **r-t**. The *p* values by two-tailed unpaired t test are indicated in **d, g-p** and **r-t**, ns, not significant, The total number of biologically independent samples are indicated in **b, d, f-p** and **r-t.** Scale bars: 10 μm (**e**), 100 μm (**l** and **o**). Source data are provided as a Source Data file.

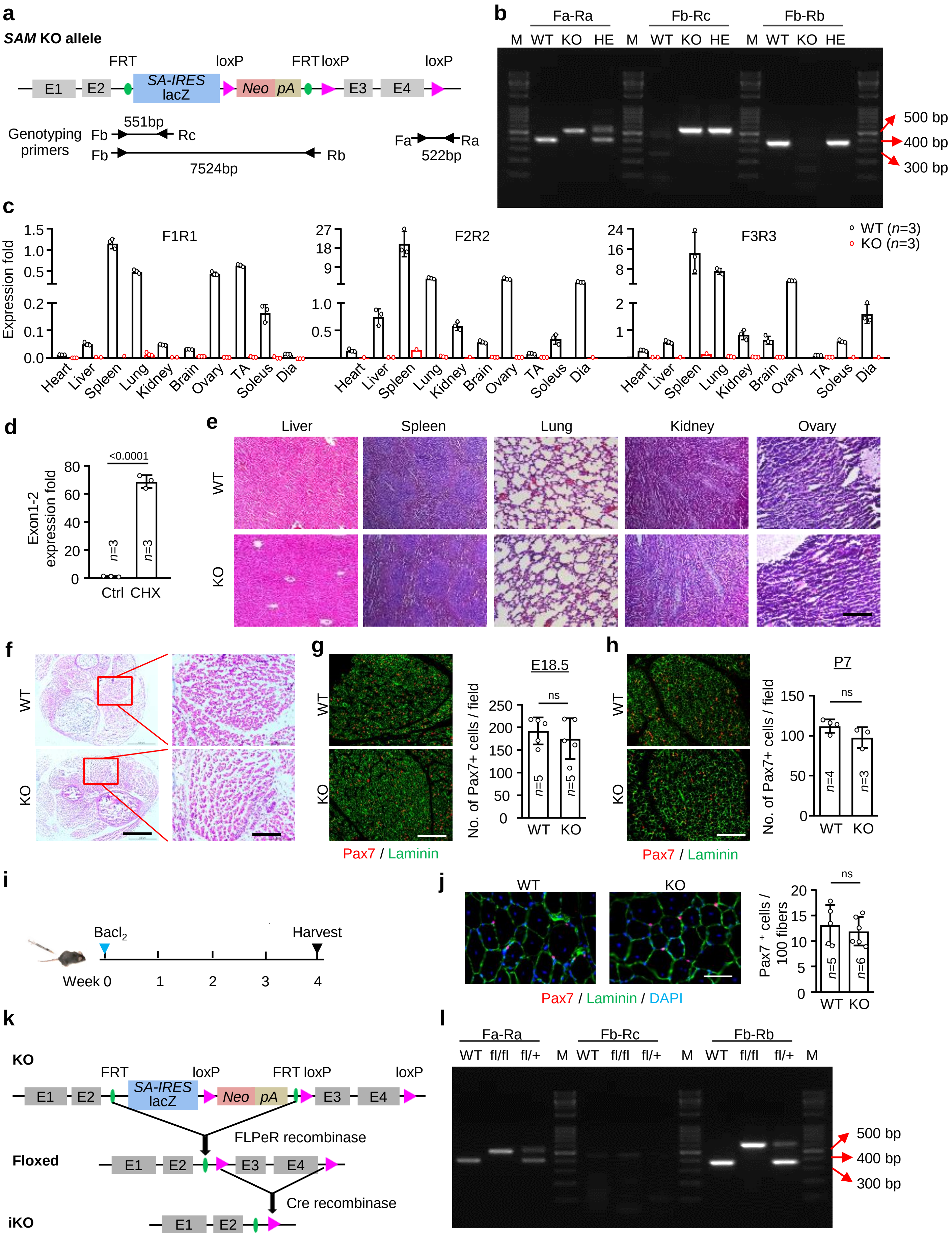

a
SAM KO allele
FRT
loxP
FRT loxP
loxP
E1
E2
SA-IRES lacZ
Neo
pA
E3
E4
Genotyping primers
Fb
Rc
551bp
Fb
Rb
7524bp
Fa
Ra
522bp
b
Fa-Ra
Fb-Rc
Fb-Rb
M WT KO HE M WT KO HE M WT KO HE
500 bp
400 bp
300 bp
c
Expression fold
F1R1
F2R2
F3R3
WT (n=3)
KO (n=3)
Heart Liver Spleen Lung Kidney Brain Ovary TA Soleus Dia
d
Exon1-2 expression fold
<0.0001
n=3
n=3
Ctrl CHX
e
Liver
Spleen
Lung
Kidney
Ovary
WT
KO
f
WT
KO
g
E18.5
WT
KO
No. of Pax7+ cells / field
ns
n=5
n=5
WT KO
Pax7 / Laminin
h
P7
WT
KO
No. of Pax7+ cells / field
ns
n=4
n=3
WT KO
Pax7 / Laminin
i
Bacl2
Harvest
Week 0 1 2 3 4
j
WT
KO
Pax7 / Laminin / DAPI
Pax7 + cells / 100 fibers
ns
n=5
n=6
WT KO
k
KO
FRT
loxP
FRT loxP
loxP
E1
E2
SA-IRES lacZ
Neo
pA
E3
E4
FLPeR recombinase
Floxed
Cre recombinase
iKO
l
Fa-Ra
Fb-Rc
Fb-Rb
WT fl/fl fl/+ M WT fl/fl fl/+ M WT fl/fl fl/+ M
500 bp
400 bp
300 bp

Supplementary Figure 2. Ablation of *SAM* in mice did not cause overt morphological abnormality. (**a**) Schematic illustration of knockout-first (KO) alleles. Arrows indicated the locations of primers used for genotyping. SA, splice acceptor; IRES, internal ribosome entry site; Neo, *Neomycin*; pA, polyadenylation signal. (**b**) Results of genotyping PCRs of WT, KO or heterozygous (HE) mice. M: DNA marker. (**c**) qRT-PCR detection of *SAM* with the above three sets of primers in major tissues of KO and WT mice. TA: Tibialis Anterior; Dia: Diaphragm. (**d**) qRT-PCR detection of transcript from exons 1-2 of *SAM* in KO ASC after CHX treatment for 6h. (**e**) H&E staining of liver, spleen, lung, kidney and ovary tissues revealed no evident abnormality in KO vs WT mice. (**f**) Representative H&E images of hindlimb muscles from WT or KO embryos at E18.5 day. Scale bar = 400μm (left) or 100μm (right). (**g**) IF staining for Pax7 and Laminin was performed in the above muscles. The numbers of Pax7+ muscle progenitor cells were quantified. (**h**) Representative images of Pax7 and Laminin staining of hindlimb muscles from WT or KO mice at 7 postnatal day. The numbers of Pax7+ muscle progenitor cells were quantified. (**i**) The scheme for $BaCl_2$ injection into TA muscle of WT or KO mice and the tissue harvesting 4 weeks afterwards for subsequent analysis. (**j**) IF staining for Pax7 and Laminin was performed in the above muscles. Quantification of Pax7+ SCs was performed. (**k**) Schematic illustration of the generation of inducible *SAM* knockout mice (*SAM* iKO). (**l**) Results of genotyping PCRs of WT, *SAM* floxed (fl/fl) and floxed heterozygous (fl/+) mice. The data are represented as mean ± SD in **c, d, g, h** and **j**. The *p* values by two-tailed unpaired t test are indicated in **d, g, h** and **j**, ns, not significant. The total number of mice used are indicated in **c, d, g, h** and **j.** Scale bars: 100 μm (**e, g** and **h**), 50 μm (**j**). Source data are provided as a Source Data file.

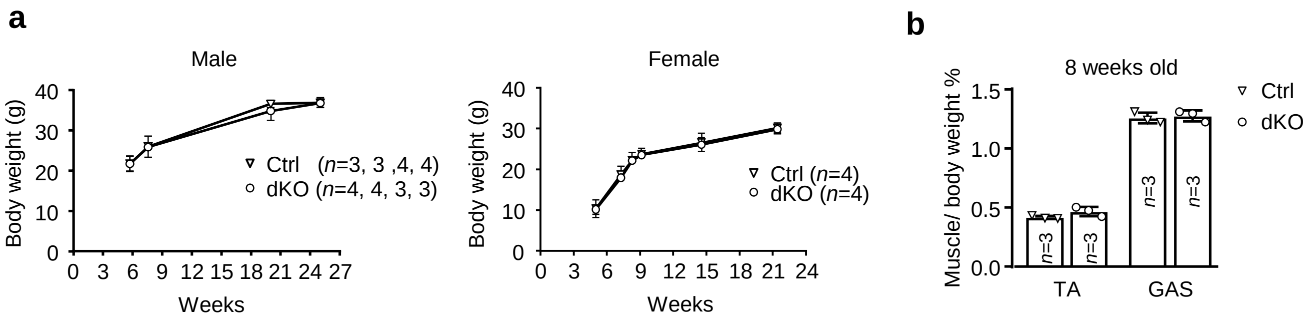

Supplementary Figure 3. Deletion of *SAM* in mdx did not affect mouse body and muscle weight. (**a**) Body weights of male (left) and female (right) mdx (Ctrl) and dKO mice were compared. (**b**) TA and Gastrocnemius (GAS) muscle weight to body weight ratio in 8-week-old female mdx and dKO mice. The data are represented as mean ± SD and the total number of mice used are indicated in **a** and **b**. Source data are provided as a Source Data file.

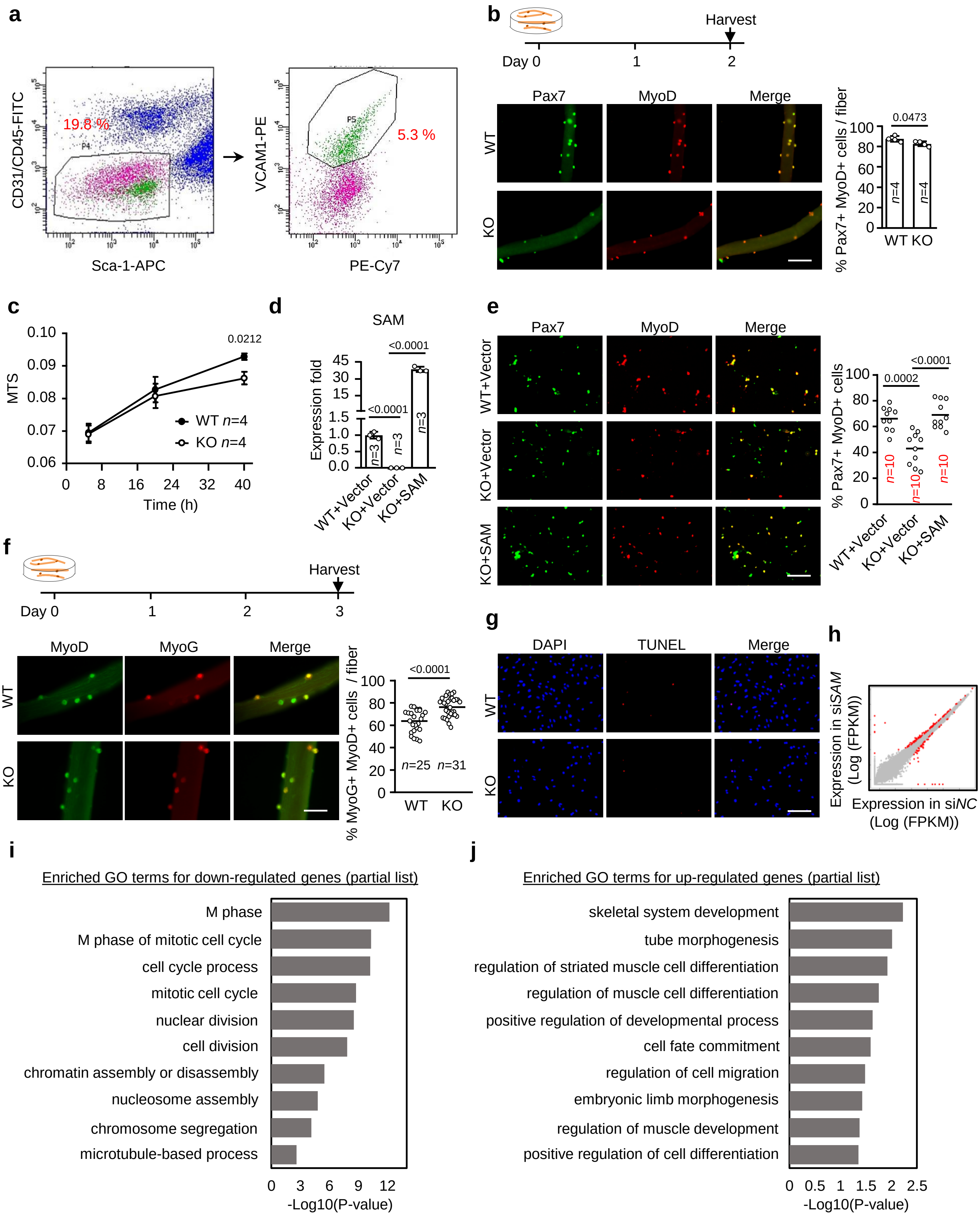
a
CD31/CD45-FITC
Sca-1-APC
19.8 %
P4
VCAM1-PE
PE-Cy7
P5
5.3 %
b
Harvest
Day 0
1
2
Pax7
MyoD
Merge
WT
KO
% Pax7+ MyoD+ cells / fiber
0.0473
n=4
n=4
WT KO
c
MTS
Time (h)
0.0212
WT n=4
KO n=4
d
SAM
Expression fold
<0.0001
<0.0001
n=3
n=3
n=3
WT+Vector
KO+Vector
KO+SAM
e
Pax7
MyoD
Merge
WT+Vector
KO+Vector
KO+SAM
% Pax7+ MyoD+ cells
0.0002
<0.0001
n=10
n=10
n=10
f
Harvest
Day 0
1
2
3
MyoD
MyoG
Merge
WT
KO
% MyoG+ MyoD+ cells / fiber
<0.0001
n=25
n=31
g
DAPI
TUNEL
Merge
WT
KO
h
Expression in siSAM (Log (FPKM))
Expression in siNC (Log (FPKM))
i
Enriched GO terms for down-regulated genes (partial list)
M phase
M phase of mitotic cell cycle
cell cycle process
mitotic cell cycle
nuclear division
cell division
chromatin assembly or disassembly
nucleosome assembly
chromosome segregation
microtubule-based process
-Log10(P-value)
j
Enriched GO terms for up-regulated genes (partial list)
skeletal system development
tube morphogenesis
regulation of striated muscle cell differentiation
regulation of muscle cell differentiation
positive regulation of developmental process
cell fate commitment
regulation of cell migration
embryonic limb morphogenesis
regulation of muscle development
positive regulation of cell differentiation
-Log10(P-value)

Supplementary Figure 4. Loss of *SAM* delayed cell proliferation and caused precocious differentiation. (**a**) Gating strategy used for FACS sorting of SCs. Sca-1, CD45, and CD31 were used as negative marker for SCs, whereas Vcam1 was used as positive marker for SCs. (**b**) Single myofibers isolated from WT and KO mice were cultured for 2 days, followed by IF staining for Pax7 and MyoD. Quantification of the percentage of double positively stained cells per myofiber was performed. At least 30 single myofibers from each mouse were examined. (**c**) FISCs from WT and KO mice were cultured for the indicated times and the proliferation ability was evaluated by MTS assay. (**d**) *SAM* expression in SCs from WT and KO mice transfected with a Vector or *SAM* expressing plasmid. (**e**) Pax7 and MyoD staining was performed in the above cells and the percentage of double positively stained cells was quantified. (**f**) Single myofibers isolated from WT and KO mice were cultured for 3 days, followed by IF for MyoD and MyoG. Quantification of the percentage of MyoD+MyoG+ cells was performed from the indicated number of myofibers. (**g**) FISCs from WT or KO mice were cultured for 48hrs and TUNEL assay was performed, showing no evident apoptosis in KO or WT cells. (**h**) *SAM* was knocked down in C2C12 myoblasts with siRNA oligos and RNA-seq was performed to assess the transcriptomic changes. The scatter plot represents differentially expressed genes in si*SAM* vs si*NC* cells. Red dots indicate genes with significant expression change (>1.5-fold). Grey dots represent genes with no differential expression. (**i-j**) GO analysis of significantly down-or up-regulated genes (partial list). The data are represented as mean ± SD in **b, d, e**, **f** and mean ± SEM in **c**. The *p* values by two-tailed unpaired t test are indicated in **b, c, d, e** and **f**. The total number of mice used in **b, c** and biologically independent samples in **d-f** are indicated. Scale bars: 100 µm (**b, e** and **g**), 50 µm (**f)**. Source data are provided as a Source Data file.

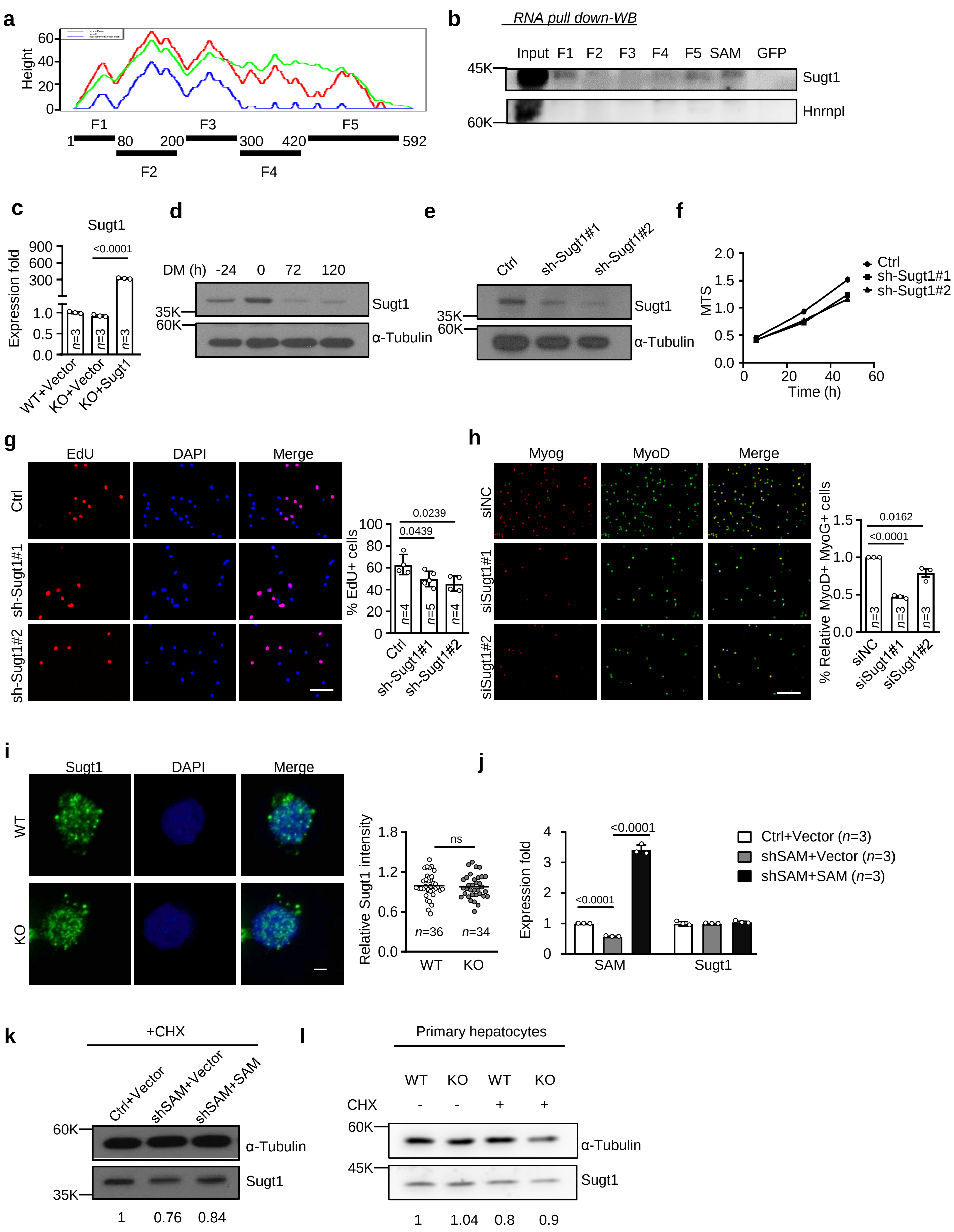

a
Height
F1
F2
F3
F4
F5
1
80
200
300
420
592
b
RNA pull down-WB
Input F1 F2 F3 F4 F5 SAM GFP
45K
60K
Sugt1
Hnrnpl
c
Sugt1
Expression fold
<0.0001
WT+Vector
KO+Vector
KO+Sugt1
d
DM (h) -24 0 72 120
35K
60K
Sugt1
α-Tubulin
e
Ctrl
sh-Sugt1#1
sh-Sugt1#2
Sugt1
α-Tubulin
f
MTS
Time (h)
Ctrl
sh-Sugt1#1
sh-Sugt1#2
g
EdU
DAPI
Merge
% EdU+ cells
0.0239
0.0439
h
Myog
MyoD
Merge
siNC
siSugt1#1
siSugt1#2
% Relative MyoD+ MyoG+ cells
0.0162
<0.0001
i
Sugt1
DAPI
Merge
WT
KO
Relative Sugt1 intensity
ns
n=36
n=34
j
Expression fold
<0.0001
<0.0001
SAM
Sugt1
Ctrl+Vector (n=3)
shSAM+Vector (n=3)
shSAM+SAM (n=3)
k
+CHX
Ctrl+Vector
shSAM+Vector
shSAM+SAM
α-Tubulin
Sugt1
1 0.76 0.84
l
Primary hepatocytes
WT KO WT KO
CHX - - + +
α-Tubulin
Sugt1
1 1.04 0.8 0.9

Supplementary Figure 5. Knockdown of Sugt1 repressed C2C12 myoblast proliferation. (**a**) Predicated secondary structure of SAM by RNAfold (http://rna.tbi.univie.ac.at/cgi-bin/RNAWebSuite/RNAfold.cgi). Top: MFE structure (Red line); the thermodynamic ensemble of RNA structures (green line); the centroid structure (blue line). Bottom: Illustration of the deletion fragments (F1-F5) of *SAM* that were used to pull down Sugt1. (**b**) Western blot (WB) analysis of the association of F1-F5 fragments or GFP transcripts with Sugt1 after RNA pull down. Association with Hnrnpl was included as a negative control. (**c**) *Sugt1* expression in ASC transfected with a Vector or Sugt1 expressing plasmid. (**d**) WB detection of Sugt1 at various time points of C2C12 differentiation. DM, differentiation medium. (**e**) Sugt1 was stably knocked down in C2C12 cells with two different shRNA oligos. (**f**) MTS assay in the above cells at different time points after seeding ($n = 4$). (**g**) The percentages of EdU+ cells were quantified in the above cells. (**h**) IF staining of MyoD and MyoG 60h after transfection of siSugt1 in SCs. The relative percentages of positively stained cells were quantified from 3 independent experiments. (**i**) SCs isolated from WT and KO mice were synchronized by nocodazole treatment for 3h followed by IF staining of Sugt1. The fluorescent signals were quantified. (**j**) A Vector or *SAM* expressing plasmid was transfected into the above Ctrl or sh*SAM* cells. The overexpression of *SAM* was confirmed 48 hrs after transfection. (**k**) WB detection of Sugt1 in the above cells with 12h-treatment of cycloheximide (CHX). The intensity of each band is shown below the blot. (**l)** WB detection of Sugt1 in primary hepatocytes isolated from WT and KO mice and treated with CHX for 10h. The data are represented as mean ± SD in **c, g, h, i**, **j** and mean ± SEM in **f**. The *p* values by two-tailed unpaired t test are indicated in **c, g, h, i** and **j,** ns, not significant. The total number of biologically independent samples in **c, f, g, i** and **j** are indicated. Scale bars: 100 μm (**g** and **h**), 2 μm (**i)**. Source data are provided as a Source Data file.

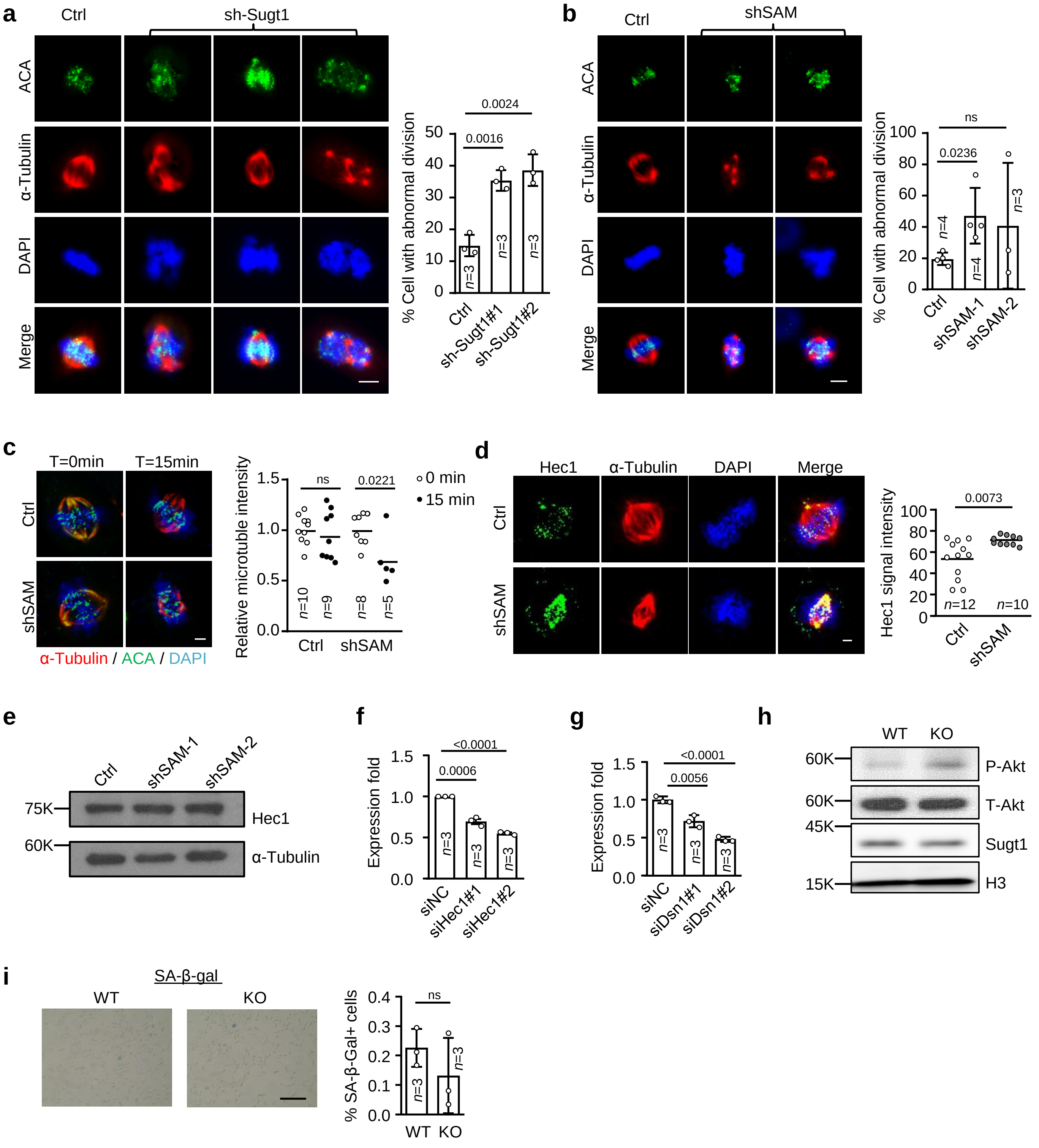
a
Ctrl
sh-Sugt1
ACA
α-Tubulin
DAPI
Merge
% Cell with abnormal division
0.0024
0.0016
n=3
Ctrl
sh-Sugt1#1
sh-Sugt1#2
b
Ctrl
shSAM
ns
0.0236
n=4
n=3
shSAM-1
shSAM-2
c
T=0min
T=15min
Ctrl
shSAM
α-Tubulin / ACA / DAPI
Relative microtubule intensity
0.0221
n=10
n=9
n=8
n=5
0 min
15 min
d
Hec1
α-Tubulin
DAPI
Merge
Hec1 signal intensity
0.0073
n=12
n=10
e
Ctrl
shSAM-1
shSAM-2
75K
60K
Hec1
α-Tubulin
f
Expression fold
<0.0001
0.0006
siNC
siHec1#1
siHec1#2
g
<0.0001
0.0056
siDsn1#1
siDsn1#2
h
WT
KO
60K
45K
15K
P-Akt
T-Akt
Sugt1
H3
i
SA-β-gal
% SA-β-Gal+ cells
WT
KO
ns
n=3

Supplementary Figure 6. *SAM*/Sugt1 facilitates kinetochore assembly in proliferating myoblasts. (**a**) IF staining for α-Tubulin and ACA in Ctrl or sh*Sugt1* C2C12 myoblasts in metaphase phase. Cells with mis-localized ACA and non-bipolarized spindle were regarded as abnormally dividing and their percentage was quantified from at least 30 cells per group from each experiment. *n*= the number of independent experiments. (**b**) The above assay was performed in Ctrl or sh*SAM* C2C12 myoblasts. At least 50 cells per group from each experiment were quantified. *n*=the number of independent experiments (**c**) Ctrl or sh*SAM* myoblasts were cold treated on ice for the indicated time and stained for α-Tubulin and ACA. The fluorescence intensity of α-Tubulin was quantified from the indicated number of cells. (**d**) IF staining of Hec1 and α-Tubulin was performed in Ctrl and sh*SAM* cells. Hec1 fluorescent signals at chromosome were quantified from the indicated number of cells. (**e**) WB detection of Hec1 expression in the above cells. (**f**) qRT-PCR detection of *Hec1* in 48h-transfected with siRNA targeting *Hec1* ASCs. (**g**) SCs were transfected with two different siRNA oligos targeting *Dsn1*. *Dsn1* knockdown was detected by qRT-PCR 2 days after transfection. (**h**) WB detection of Phospho-Akt (S473) (P-Akt), total Akt (T-Akt), Sugt1 and H3 expression in WT and KO SCs cultured for 4 days. (**i**) FACS sorted SCs from WT and KO mice were cultured for 3 days and the percentage of SA-β-Gal+ cells was quantified. The data are represented as mean ± SD in **a-d, f, g** and **i**. The *p* values by two-tailed unpaired t test are indicated in **a-d, f, g** and **i,** ns, not significant. The total number of independent experiments in **a, b, i** and biologically independent samples in **c, d**, **f** and **g** are indicated. Scale bars: 5 μm (**a** and **b**), 2 μm (**c** and **d)**, 100 μm (**i**). Source data are provided as a Source Data file.

Supplementary Table 1: Primers used for genotyping, RT-qPCR and Clone

**Genotyping**	
Name	Sequence
SAM-Fa	CTAATCAGAATACATCAGAGGCAAGA
SAM-Ra	AGGTACTGGAAGCACAAACCCT
SAM-Fb	GGGTCTCCGGTGTTGAAGTAAA
SAM-Rb	AAATGAGGGCCTGGTGTGGTAAC
SAM-Rc	CCAACTGACCTTGGGCAAGAACAT
Flpe-wild type-F	CCCAAAGTCGCTCTGAGTTGTTATC
Flpe-wild type-R	TGACTACCTATCCTCCCATTTTCCT
Flpe-mutant-F	CACTGATATTGTAAGTAGTTTGC
Flpe-mutant-R	CTAGTGCGAAGTAGTGATCAGG
Pax7-CreER-mutant	CAAAAGACGGCAATATGGTG
Pax7-CreER-wild type	CTGCACTGAGACAGGACCG
Pax7-CreER-Common	GCTGCTGTTGATTACCTGGC
EYFP-mutant	AAGACCGCGAAGAGTTTGTC
EYFP-wild type	GGAGCGGGAGAAATGGATATG
EYFP-Common	AAAGTCGCTCTGAGTTGTTAT
nGFP-R	CACATGAAGCAGCACGACTT
nGFP-F	TGCTCAGGTAGTGGTTGTCG
**qRT-PCR**	
Name	Sequence
SAM-F1	GCCCTCACCTTATCTTCCGT
SAM-R1	AAGCTCGGTCATCCCTTCCA
SAM-F2	TACAGCGTGGAAGGGATGAC
SAM-R2	CACGAAACAGCCTGATGGTC
SAM-F3	GAAACCTAAGGTGCTGGAGCTAGA
SAM-R3	CGGATCCCCATTACAGATAATTGT
Sugt1-F	CCGTGATGGTATTGCCGATGT
Sugt1-R	GGCAGAAGCGTAGTCTTTTTCA
GAPDH-F	AGAACATCATCCCTGCATCC
GAPDH-R	GGTCCTCAGTGTAGCCCAAG
18S-F	GTAACCCGTTGAACCCCATT
18S-R	CCATCCAATCGGTAGTAGCG
PAX7-F	GCTACCAGTACAGCCAGTATG
PAX7-R	GTCACTAAGCATGGGTAGATG
MyoD-F	CTGCTCTGATGGCATGATGG
MyoD-R	GTTCCCTGTTCTGTGTCGCT
DUM-F	GGGATGCGAGTCTCCTCTTG
DUM-R	GACGATCATTCGCTTGACTTTG
a-Actin-F	CACCAGGGTGTCATGGTAGG
a-Actin-R	TGGTACGGCCGGAAGCATAG
Malat1-F	TTTGCATTGGACTTGAGCTG
Malat1-R	TGGCTCCTCAGTCCTTCCTA
Dsn1-RT-F1	TGTCCGAGACTCAGGATCGTC
Dsn1-RT-R1	GTTCGAGCCAAGGTGGTTTTT
HEC1-RT-F	GCCTCTCTATGCAGGAGTTAAGG
HEC1-RT-R	CGGTTTGTGTGTACTCAGCTT
**Clone**	
Name	Sequence
SAM-Full-F	AGTGCTAGCAACTTTAAGCACACGTGAGCGTCCTC
SAM-full-R	AGTGGTACCTGGAGTCTCATTTCTATTTATTAATTTAC
Flag-Sugt1 pcDNA-F	CGGGGTACCATGGATTACAAGGATGACGACGATAAGGCGGCAGCTGCAGCAGGACC
Flag-Sugt1 PcDNA-R	TGCTCTAGATTAGTACTGTTTCCATTCCA
sh-sugt1-1-Top	GATCCGCCGGATGACGCACAGTATTTTCAAGAGAAATACTGTGCGTCATCCGGCTTTTTTG
sh-sugt1-1-Bottom	AATTCAAAAAAGCCGGATGACGCACAGTATTTCTCTTGAAAATACTGTGCGTCATCCGGCG
sh-sugt1-2-Top	GATCCGCCGAGTCTCACGTAATCATTTCAAGAGAATGATTACGTGAGACTCGGCTTTTTTG
sh-sugt1-2-Bottom	AATTCAAAAAAGCCGAGTCTCACGTAATCATTCTCTTGAAATGATTACGTGAGACTCGGCG
sh-SAM-1-Top	GATCCGGAGATGACTCAACTGTTAATTCAAGAGATTAACAGTTGAGTCATCTCCTTTTTTG
sh-SAM-1-Bottom	AATTCAAAAAAGGAGATGACTCAACTGTTAATCTCTTGAATTAACAGTTGAGTCATCTCCG
sh-SAM-2-Top	GATCCGGTGATGGTGTACTGACATATTCAAGAGATATGTCAGTACACCATCACCTTTTTTG
sh-SAM-2-Bottom	AATTCAAAAAAGGTGATGGTGTACTGACATATCTCTTGAATATGTCAGTACACCATCAC

Supplementary Table 2: Oligonucleotides used for siRNA and FISH probe

Name	Sequence
**siRNA**	
Name	Sequence
Si-SAM-1-Sense	GAGAUGACUCAACUGUUAA
Si-SAM-1-Antisense	UUAACAGUUGAGUCAUCUC
Si-SAM-2-Sense	GUGAUGGUGUACUGACAUA
Si-SAM-2-Antisense	UAUGUCAGUACACCAUCAC
Si-Sugt1#1-Sense	CCGGAUGACGCACAGUAUU
Si-Sugt1#1-Antisense	AAUACUGUGCGUCAUCCGG
Si-Sugt1#2-Sense	CCGAGUCUCACGUAAUCAU
Si-Sugt1#2-Antisense	AUGAUUACGUGAGACUCGG
siDsn1#1-Sense	CCAGGAAAUGAUGACGCAATT
siDsn1#1-Antisense	UUGCGUCAUCAUUUCCUGGTT
siDsn1#2-Sense	GGAGACGAGCAAGCAUGAATT
siDsn1#2-Antisense	UUCAUGCUUGCUCGUCUCCTT
siHec1#1-Sense	GCAAGCUCCAUCCACUAAATT
siHec1#1-Antisense	UUUAGUGGAUGGAGCUUGCTT
siHec1#2-Sense	GCAACUUGGAGUCUCAUUUTT
siHec1#2-Antisense	AAAUGAGACUCCAAGUUGCTT
**FISH probe**	
Name	Sequence
SAM-FISH-1	ACGCTCACGTGTGCTTAAAG
SAM-FISH-2	CAGCACATGCTAGGAAGGAC
SAM-FISH-3	ACGGAAGATAAGGTGAGGGC
SAM-FISH-4	TCTACCTGGACTCGGAATAG
SAM-FISH-5	TTAGGTTTCCAGGACATGTC
SAM-FISH-6	GTGCTATCAAGTCTCTTCTA
SAM-FISH-7	AAACAGCCTGATGGTCTCTG
SAM-FISH-8	TCTCTTTGGTGGAAGTTCCA
SAM-FISH-9	CTCTGGAGTCTCCATTTCTG
