# Supplementary material for: Long noncoding RNA SAM promotes myoblast proliferation through stabilizing Sugt1 and facilitating kinetochore assembly
Source: Nat Commun. 2020 Jun 1;11:2725. doi: 10.1038/s41467-020-16553-6 (PMC7264179; doi:10.1038/s41467-020-16553-6)
Supplement: Supplementary file 3 — Reporting Summary [file 41467_2020_16553_MOESM3_ESM.pdf]

nature research

Corresponding author(s): Huating Wang

Last updated by author(s): 2020-3-29

Reporting Summary

Nature Research wishes to improve the reproducibility of the work that we publish. This form provides structure for consistency and transparency in reporting. For further information on Nature Research policies, see Authors & Referees and the Editorial Policy Checklist .

Statistics

For all statistical analyses, confirm that the following items are present in the figure legend, table legend, main text, or Methods section.

n/a	Confirmed	
☐	☒	The exact sample size (n) for each experimental group/condition, given as a discrete number and unit of measurement
☐	☒	A statement on whether measurements were taken from distinct samples or whether the same sample was measured repeatedly
☐	☒	The statistical test(s) used AND whether they are one- or two-sided<br>*Only common tests should be described solely by name; describe more complex techniques in the Methods section.*
☒	☐	A description of all covariates tested
☒	☐	A description of any assumptions or corrections, such as tests of normality and adjustment for multiple comparisons
☐	☒	A full description of the statistical parameters including central tendency (e.g. means) or other basic estimates (e.g. regression coefficient) AND variation (e.g. standard deviation) or associated estimates of uncertainty (e.g. confidence intervals)
☐	☒	For null hypothesis testing, the test statistic (e.g. F, t, r) with confidence intervals, effect sizes, degrees of freedom and P value noted<br>*Give P values as exact values whenever suitable.*
☒	☐	For Bayesian analysis, information on the choice of priors and Markov chain Monte Carlo settings
☒	☐	For hierarchical and complex designs, identification of the appropriate level for tests and full reporting of outcomes
☒	☐	Estimates of effect sizes (e.g. Cohen's d, Pearson's r), indicating how they were calculated

Our web collection on statistics for biologists contains articles on many of the points above.

Software and code

Policy information about availability of computer code

Data collection	qPCR reactions were conducted using the Light Cycler ®480 Real-Time PCR System (Roche Applied Science);<br>Microscopy pictures were acquired with Leica microscope system DM 6000B and laser scanning confocal microscope (Carl ZEISS LSM 880); BD FACSVerse flow cytometer, BD FACSAria Fusion Cell Sorter and BD FACSDiva (version 8.0.1, BD Biosciences) were used to acquisition of flow cytometry data.
Data analysis	GraphPad Prism version 8.0 was used to data analysis.<br>Leica LAS AF software (LAS AF2.6.3) was used to acquisition and analysis of images from Leica microscope.<br>ZEN 2.3 (blue edition) software was used to acquisition of images from confocal microscope.<br>ImageJ 1.50i (National Institutes of Health) were used to Western blot band intensities quantification.<br>WinMDI 2.8. and BD FACSDiva 8.0.1 were used to acquire and analyze flow cytometry data.<br>Measurements of tubulin and Hec1, Dsn1 and Sugt1 intensities were conducted by in house program written in MATLAB (R2014b) language.<br>Measurements of Collagen 1 and collagen positive area were conducted by in house ImageCount software written in MATLAB (R2014b) language.<br>MATLAB (R2014b) language codes used in this study have been deposited on GitHub (https://github.com/jieyuanCUHK/SAM_paper).

For manuscripts utilizing custom algorithms or software that are central to the research but not yet described in published literature, software must be made available to editors/reviewers. We strongly encourage code deposition in a community repository (e.g. GitHub). See the Nature Research guidelines for submitting code & software for further information.

Data

Policy information about availability of data

All manuscripts must include a data availability statement. This statement should provide the following information, where applicable:

- Accession codes, unique identifiers, or web links for publicly available datasets
- A list of figures that have associated raw data
- A description of any restrictions on data availability

Data availability
RNA-seq data have been deposited in the Gene Expression Omnibus under the accession code GSE126423. The mass spectrometry proteomics data have been deposited to the ProteomeXchange Consortium via the PRIDE57 partner repository with the dataset identifier PXD018147. The source data underlying Figs. 1c-e, 1g-n, 2b, d, f, h-k, n, p, 3b, d, f, g, i, j, 4a-l, 5a, c, d, f-o, 6a-h and Supplementary Figs. 1b, d, f-t, 2b-d, g, h, j, l, 3a, b, 4b-f, 5b-l, 6a-i are provided in the Source Data file. All other data supporting the findings of this study are available from the corresponding author on reasonable request. A reporting summary for this article is available as a Supplementary Information file.
Code availability
MATLAB language codes used in this study have been deposited on GitHub (https://github.com/jieyuanCUHK/SAM_paper).

The proteomics data are currently private, reviewer account details as below:
Username: reviewer10657@ebi.ac.uk
Password: EJ1jE4dz

Field-specific reporting

Please select the one below that is the best fit for your research. If you are not sure, read the appropriate sections before making your selection.

- [x] Life sciences
- [ ] Behavioural & social sciences
- [ ] Ecological, evolutionary & environmental sciences

For a reference copy of the document with all sections, see nature.com/documents/nr-reporting-summary-flat.pdf

Life sciences study design

All studies must disclose on these points even when the disclosure is negative.

Sample size	At least three biological replicates per group (detailed n is indicated in the figure or figure legends) were collected to perform statistical testing. For RNA-seq, one biological replicate per group were used. These sample sizes in Figure 6a-6e and 6h and Supplementary figure 5i, 6a-6d are sufficient basing on previous studies. Sample size of each group of mice was determined to be adequate based on the magnitude and consistency of measurable differences between groups.
Data exclusions	No data or samples were excluded from the analysis
Replication	All experimental data was repeated in multiple biological independent experiments as indicated in the legend, method and source data.
Randomization	For all the animal experiments, we used the the same age and sex of WT and KO mice from the same litter whenever possible. For cell experiments, we randomly counted multiple fields or cells per group for calculation.
Blinding	For immunofluorescence data collection , we performed the experiments in a blinded way. We randomly counted multiple fields per group and calculated the number of positively stained cells per field. For immunofluorescence intensity quantification, we were blinded to cell allocation during data analysis. For RNA analyses using qRT-PCR and Protein analyses using Western blotting were not performed blind to load samples by order.

Behavioural & social sciences study design

All studies must disclose on these points even when the disclosure is negative.

Study description	*Briefly describe the study type including whether data are quantitative, qualitative, or mixed-methods (e.g. qualitative cross-sectional, quantitative experimental, mixed-methods case study).*
Research sample	*State the research sample (e.g. Harvard university undergraduates, villagers in rural India) and provide relevant demographic information (e.g. age, sex) and indicate whether the sample is representative. Provide a rationale for the study sample chosen. For studies involving existing datasets, please describe the dataset and source.*
Sampling strategy	*Describe the sampling procedure (e.g. random, snowball, stratified, convenience). Describe the statistical methods that were used to predetermine sample size OR if no sample-size calculation was performed, describe how sample sizes were chosen and provide a rationale for why these sample sizes are sufficient. For qualitative data, please indicate whether data saturation was considered, and what criteria were used to decide that no further sampling was needed.*

Data collection	*Provide details about the data collection procedure, including the instruments or devices used to record the data (e.g. pen and paper, computer, eye tracker, video or audio equipment) whether anyone was present besides the participant(s) and the researcher, and whether the researcher was blind to experimental condition and/or the study hypothesis during data collection.*
Timing	*Indicate the start and stop dates of data collection. If there is a gap between collection periods, state the dates for each sample cohort.*
Data exclusions	*If no data were excluded from the analyses, state so OR if data were excluded, provide the exact number of exclusions and the rationale behind them, indicating whether exclusion criteria were pre-established.*
Non-participation	*State how many participants dropped out/declined participation and the reason(s) given OR provide response rate OR state that no participants dropped out/declined participation.*
Randomization	*If participants were not allocated into experimental groups, state so OR describe how participants were allocated to groups, and if allocation was not random, describe how covariates were controlled.*

Ecological, evolutionary & environmental sciences study design

All studies must disclose on these points even when the disclosure is negative.

Study description	*Briefly describe the study. For quantitative data include treatment factors and interactions, design structure (e.g. factorial, nested, hierarchical), nature and number of experimental units and replicates.*
Research sample	*Describe the research sample (e.g. a group of tagged Passer domesticus, all Stenocereus thurberi within Organ Pipe Cactus National Monument), and provide a rationale for the sample choice. When relevant, describe the organism taxa, source, sex, age range and any manipulations. State what population the sample is meant to represent when applicable. For studies involving existing datasets, describe the data and its source.*
Sampling strategy	*Note the sampling procedure. Describe the statistical methods that were used to predetermine sample size OR if no sample-size calculation was performed, describe how sample sizes were chosen and provide a rationale for why these sample sizes are sufficient.*
Data collection	*Describe the data collection procedure, including who recorded the data and how.*
Timing and spatial scale	*Indicate the start and stop dates of data collection, noting the frequency and periodicity of sampling and providing a rationale for these choices. If there is a gap between collection periods, state the dates for each sample cohort. Specify the spatial scale from which the data are taken*
Data exclusions	*If no data were excluded from the analyses, state so OR if data were excluded, describe the exclusions and the rationale behind them, indicating whether exclusion criteria were pre-established.*
Reproducibility	*Describe the measures taken to verify the reproducibility of experimental findings. For each experiment, note whether any attempts to repeat the experiment failed OR state that all attempts to repeat the experiment were successful.*
Randomization	*Describe how samples/organisms/participants were allocated into groups. If allocation was not random, describe how covariates were controlled. If this is not relevant to your study, explain why.*
Blinding	*Describe the extent of blinding used during data acquisition and analysis. If blinding was not possible, describe why OR explain why blinding was not relevant to your study.*

Did the study involve field work? ☐ Yes ☐ No

Field work, collection and transport

Field conditions	*Describe the study conditions for field work, providing relevant parameters (e.g. temperature, rainfall).*
Location	*State the location of the sampling or experiment, providing relevant parameters (e.g. latitude and longitude, elevation, water depth).*
Access and import/export	*Describe the efforts you have made to access habitats and to collect and import/export your samples in a responsible manner and in compliance with local, national and international laws, noting any permits that were obtained (give the name of the issuing authority, the date of issue, and any identifying information).*
Disturbance	*Describe any disturbance caused by the study and how it was minimized.*

Reporting for specific materials, systems and methods

We require information from authors about some types of materials, experimental systems and methods used in many studies. Here, indicate whether each material, system or method listed is relevant to your study. If you are not sure if a list item applies to your research, read the appropriate section before selecting a response.

Materials & experimental systems

n/a	Involved in the study
☐	☒ Antibodies
☐	☒ Eukaryotic cell lines
☐	☐ Palaeontology
☐	☒ Animals and other organisms
☐	☐ Human research participants
☐	☐ Clinical data

Methods

n/a	Involved in the study
☐	☐ ChIP-seq
☐	☒ Flow cytometry
☐	☐ MRI-based neuroimaging

Antibodies

Antibodies used

For Western blot, the following antibodies were used:
Mouse anti-Sugt1 (YQ-R3) (sc-81822, Santa Cruz); mouse anti-α-Tubulin (B-5-1-2) (sc-23948, Santa Cruz); mouse anti-Flag (M2) (F1804, Sigma); mouse anti-Ub (P4D1) (sc-8017, Santa Cruz); mouse anti-HA (F-7) (sc-7392, Santa Cruz); rabbit anti-hnrnpl (H-78) (sc-28726, Santa Cruz); mouse anti-Dnmt 3a [64B1446] (ab-13888, Abcam); rabbit anti-Dnmt 3b (ab-2851, Abcam) and rabbit anti-Hec1 antibody (it is a very kind gift from Dr. Robert Benezra, Memorial Sloan Kettering Cancer Center, USA),
For immunofluorescent staining, the following antibodies were used:
Rabbit anti-MyoD (M-318) (sc-760,Santa Cruz); rabbit anti-Myogenin (M-225) (sc-576, Santa Cruz); mouse anti-Pax7 (Developmental Studies Hybridoma Bank); mouse anti-MF20 (Developmental Studies Hybridoma Bank); Donkey anti-Mouse IgG (H+L) Cross-Adsorbed, Alexa Fluor® 488 (A21202, invitrogen); Donkey anti-Mouse IgG (H+L) Cross-Adsorbed, Alexa Fluor® 594 (A21203, Invitrogen), Goat anti-Rabbit IgG (H+L) Cross-Adsorbed, Alexa Fluor® 488 (A11008 Invitrogen); Donkey anti-Rabbit IgG (H+L) Highly Cross-Adsorbed, Alexa Fluor® 594 (A21207, invitrogen);mouse anti-α-Tubulin (B-5-1-2) (sc-23948, Santa Cruz); ACA (15-235-F, Antibodies Incorporated); The biotin conjugated anti-mouse IgG (115-065- 205, Jackson); Cy3-Streptavidin (016-160-084, Jackson); mouse anti-MyoD (M3512, Dako), mouse anti-eMyHC (NCL-MHC-d,Leica) and rabbit anti-laminin (L9393,Sigma-Aldrich); rabbit anti-Dsn1 (orb101208, Biorbyt); rabbit anti-Collagen1 (Novus, NBP1-30054).
For Native RNA immunoprecipitation (rip) assay, the following antibodies were used:
anti-Sugt1 (sc-81822, Santa Cruz); Normal mouse IgG (sc-2027, Santa Cruz)
For fluorescence-activated cell sorting, the following antibodies were used:
Vcam1-biotin (105704, BioLegend), CD31-FITC (102506, BioLegend), CD45-FITC (103108, BioLegend), Sca1-Alxa647 (108118, BioLegend), streptavidin-PE-cy7 (405206, BioLegend) and Streptavidin-PE (554061, BD Biosciences).

Validation

Rabbit anti-Hec1 antibody was generated in Dr. Robert Benezra's lab and its validation for the species and application was reported in ref. 9 and all other antibodies are commercially available and the applications have been tested by the manufacturer

Eukaryotic cell lines

Policy information about cell lines

Cell line source(s)

Mouse C2C12 myoblast cells (CRL-1772) were obtained from American Type Culture Collection (ATCC)

Authentication

Cell lines were procured from commercial source and therefore, not authenticated.

Mycoplasma contamination

Cell lines were tested negative for mycoplasma.

Commonly misidentified lines
(See ICLAC register)

No commonly misidentified cell lines were involved in this study

Palaeontology

Specimen provenance

Provide provenance information for specimens and describe permits that were obtained for the work (including the name of the issuing authority, the date of issue, and any identifying information).

Specimen deposition

Indicate where the specimens have been deposited to permit free access by other researchers.

Dating methods

If new dates are provided, describe how they were obtained (e.g. collection, storage, sample pretreatment and measurement), where they were obtained (i.e. lab name), the calibration program and the protocol for quality assurance OR state that no new dates are provided.

☐ Tick this box to confirm that the raw and calibrated dates are available in the paper or in Supplementary Information.

Animals and other organisms

Policy information about studies involving animals; ARRIVE guidelines recommended for reporting animal research

Laboratory animals

SAM KO heterozygote (SAM-/+) mice (C57BL/6 background) were generated in the Model Animal Research Center of the Nanjing University (Nanjing, China). FLPeR mice were purchased in the Model Animal Research Center of the Nanjing University (Nanjing,

	China). SAM KO strain (SAM KO: SAM-/-, littermate control: SAM+/+) were housed in our laboratory animal services center at the Chinese University of Hong Kong (CUHK). Pax7creER mice were purchased from the Jackson Laboratory. Mdx mouse strains were purchased from The Jackson Laboratory. Pax7-nGFP mice were gifts from by Prof. Shahragim Tajbakhsh (Institut Pasteur). SAM iKO strain (SAM-iKO: SAMfl/fl; Pax7creER/+, littermate control: SAM+/+; Pax7creER/+) were obtained by crossing SAM KO mice with FLPeR mice and Pax7creER mice. All mice were maintained in animal room with 12 h light/12 h dark cycles, temperature (22–24 خ), humidity (40-60%) at animal facility in The Chinese University of Hong Kong.
Wild animals	The study did not involve wild animals.
Field-collected samples	The study did not involve samples collected from the field.
Ethics oversight	All animal experiments were performed in accordance with guidelines approved by the Animal Experimentation Ethics Committee of the Chinese University of Hong Kong (Ref No. 15/027/MIS-6-U).

Note that full information on the approval of the study protocol must also be provided in the manuscript.

Human research participants

Policy information about studies involving human research participants

Population characteristics	*Describe the covariate-relevant population characteristics of the human research participants (e.g. age, gender, genotypic information, past and current diagnosis and treatment categories). If you filled out the behavioural & social sciences study design questions and have nothing to add here, write "See above."*
Recruitment	*Describe how participants were recruited. Outline any potential self-selection bias or other biases that may be present and how these are likely to impact results.*
Ethics oversight	*Identify the organization(s) that approved the study protocol.*

Note that full information on the approval of the study protocol must also be provided in the manuscript.

Clinical data

Policy information about clinical studies

All manuscripts should comply with the ICMJEguidelines for publication of clinical research and a completedCONSORT checklist must be included with all submissions.

Clinical trial registration	*Provide the trial registration number from ClinicalTrials.gov or an equivalent agency.*
Study protocol	*Note where the full trial protocol can be accessed OR if not available, explain why.*
Data collection	*Describe the settings and locales of data collection, noting the time periods of recruitment and data collection.*
Outcomes	*Describe how you pre-defined primary and secondary outcome measures and how you assessed these measures.*

ChIP-seq

Data deposition

☐ Confirm that both raw and final processed data have been deposited in a public database such as GEO.

☐ Confirm that you have deposited or provided access to graph files (e.g. BED files) for the called peaks.

Data access links<br>*May remain private before publication.*	*For "Initial submission" or "Revised version" documents, provide reviewer access links. For your "Final submission" document, provide a link to the deposited data.*
Files in database submission	*Provide a list of all files available in the database submission.*
Genome browser session<br>(e.g. UCSC)	*Provide a link to an anonymized genome browser session for "Initial submission" and "Revised version" documents only, to enable peer review. Write "no longer applicable" for "Final submission" documents.*

Methodology

Replicates	*Describe the experimental replicates, specifying number, type and replicate agreement.*
Sequencing depth	*Describe the sequencing depth for each experiment, providing the total number of reads, uniquely mapped reads, length of reads and whether they were paired- or single-end.*
Antibodies	*Describe the antibodies used for the ChIP-seq experiments; as applicable, provide supplier name, catalog number, clone name, and lot number.*

Peak calling parameters

Specify the command line program and parameters used for read mapping and peak calling, including the ChIP, control and index files used.

Data quality

Describe the methods used to ensure data quality in full detail, including how many peaks are at FDR 5% and above 5-fold enrichment.

Software

Describe the software used to collect and analyze the ChIP-seq data. For custom code that has been deposited into a community repository, provide accession details.

Flow Cytometry

Plots

Confirm that:

- [x] The axis labels state the marker and fluorochrome used (e.g. CD4-FITC).
- [x] The axis scales are clearly visible. Include numbers along axes only for bottom left plot of group (a 'group' is an analysis of identical markers).
- [x] All plots are contour plots with outliers or pseudocolor plots.
- [x] A numerical value for number of cells or percentage (with statistics) is provided.

Methodology

Sample preparation

Muscle satellite cells preparation is included in the "Methods" section. 20,000 events were recorded and are shown in the plots.
PI/FACS (cell cycle) analysis:
Harvest cells and wash with PBS once. Suspend cells in 500ul of PBS containing 0.1% Glucose and immediately add 5 ml of cold 70% ETOH. Mix immediately and keep at 4 خ for overnight. Spin cells down and wash with PBS. Suspend in 300ul propidium iodide solution and add 20ul of 10mg/ml RNase. Mix and incubate at 37خ for 45min. Transfer to FACS tubes and run samples on flow cytometer and approximately 10,000 events were analysed for each sample. For live cell-cycle analysis, myoblasts were labeled with Hoechst 33342 (5ug/ml) for 45min at 37 °C and sorted by BD FACSAria Fusion Cell Sorter. 20,000 events were recorded and are shown in the plots.

Instrument

BD FACSAria Fusion Cell Sorter and BD FASC Calibur

Software

WinMDI 2.8. and BD FACSDiva

Cell population abundance

Muscle satellite cells sorting
We stained isolated cells by FACS with Pax7 antibody which is canonical marker of satellite cells and found 98% cells were Pax7 positive cells.

Gating strategy

Muscle satellite cells sorting
Gating strategy was done according to previously reported protocol PMID: 26401916
PI/FACS (cell cycle) analysis:
Set appropriate FSC vs SSC gates to exclude debris and cell aggregates. Collect Propidium iodide fluorescence in FL2, or Hochest

- [x] Tick this box to confirm that a figure exemplifying the gating strategy is provided in the Supplementary Information.

Magnetic resonance imaging

Experimental design

Design type

Indicate task or resting state; event-related or block design.

Design specifications

Specify the number of blocks, trials or experimental units per session and/or subject, and specify the length of each trial or block (if trials are blocked) and interval between trials.

Behavioral performance measures

State number and/or type of variables recorded (e.g. correct button press, response time) and what statistics were used to establish that the subjects were performing the task as expected (e.g. mean, range, and/or standard deviation across subjects).

Acquisition

Imaging type(s)	*Specify: functional, structural, diffusion, perfusion.*
Field strength	*Specify in Tesla*
Sequence & imaging parameters	*Specify the pulse sequence type (gradient echo, spin echo, etc.), imaging type (EPI, spiral, etc.), field of view, matrix size, slice thickness, orientation and TE/TR/flip angle.*
Area of acquisition	*State whether a whole brain scan was used OR define the area of acquisition, describing how the region was determined.*

Diffusion MRI ☐ Used ☐ Not used

Preprocessing

Preprocessing software	*Provide detail on software version and revision number and on specific parameters (model/functions, brain extraction, segmentation, smoothing kernel size, etc.).*
Normalization	*If data were normalized/standardized, describe the approach(es): specify linear or non-linear and define image types used for transformation OR indicate that data were not normalized and explain rationale for lack of normalization.*
Normalization template	*Describe the template used for normalization/transformation, specifying subject space or group standardized space (e.g. original Talairach, MNI305, ICBM152) OR indicate that the data were not normalized.*
Noise and artifact removal	*Describe your procedure(s) for artifact and structured noise removal, specifying motion parameters, tissue signals and physiological signals (heart rate, respiration).*
Volume censoring	*Define your software and/or method and criteria for volume censoring, and state the extent of such censoring.*

Statistical modeling & inference

Model type and settings	*Specify type (mass univariate, multivariate, RSA, predictive, etc.) and describe essential details of the model at the first and second levels (e.g. fixed, random or mixed effects; drift or auto-correlation).*
Effect(s) tested	*Define precise effect in terms of the task or stimulus conditions instead of psychological concepts and indicate whether ANOVA or factorial designs were used.*

Specify type of analysis: ☐ Whole brain ☐ ROI-based ☐ Both

Statistic type for inference<br>(See Eklund et al. 2016)	*Specify voxel-wise or cluster-wise and report all relevant parameters for cluster-wise methods.*
Correction	*Describe the type of correction and how it is obtained for multiple comparisons (e.g. FWE, FDR, permutation or Monte Carlo).*

Models & analysis

n/a	Involved in the study
☐	☐ Functional and/or effective connectivity
☐	☐ Graph analysis
☐	☐ Multivariate modeling or predictive analysis

Functional and/or effective connectivity	*Report the measures of dependence used and the model details (e.g. Pearson correlation, partial correlation, mutual information).*
Graph analysis	*Report the dependent variable and connectivity measure, specifying weighted graph or binarized graph, subject- or group-level, and the global and/or node summaries used (e.g. clustering coefficient, efficiency, etc.).*
Multivariate modeling and predictive analysis	*Specify independent variables, features extraction and dimension reduction, model, training and evaluation metrics.*
