# Supplementary material for: Long noncoding RNA SAM promotes myoblast proliferation through stabilizing Sugt1 and facilitating kinetochore assembly
Source: Nat Commun. 2020 Jun 1;11:2725. doi: 10.1038/s41467-020-16553-6 (PMC7264179; doi:10.1038/s41467-020-16553-6)
Supplement: Supplementary file 4 — Description of Additional Supplementary Files [file 41467_2020_16553_MOESM4_ESM.pdf]

Description of Additional Supplementary Files

File Name: Supplementary Movie 1
Description: The 3D z-stack confocal images for α-Tubulin (Red) and DAPI (Blue) staining in ASCs from KO.

File Name: Supplementary Movie 2
Description: The 3D z-stack confocal images for α-Tubulin (Red) and DAPI (Blue) staining in ASCs from KO.

File Name: Supplementary Movie 3
Description: The 3D z-stack confocal images for α-Tubulin (Red) and DAPI (Blue) staining in ASCs from KO.

File Name: Supplementary Movie 4
Description: The 3D z-stack confocal images for α-Tubulin (Red) and DAPI (Blue) staining in ASCs from WT.
